# Effect of melatonin on gut microbiome and metabolomics in diabetic cognitive impairment

**DOI:** 10.3389/fphar.2024.1489834

**Published:** 2024-11-21

**Authors:** Ming Gao, Jie Li, Xu Han, Beiyao Zhang, Jinting Chen, Jiadong Lang, Qiangqiang Zhang

**Affiliations:** ^1^ Department of Endocrinology and Rare Disease, The Second Hospital of Hebei Medical University, Shijiazhuang, Hebei, China; ^2^ Hebei Key Laboratory of Rare Disease, Hebei Provincial Department of Science and Technology, Shijiazhuang, Hebei, China; ^3^ Core Facilities and Centers, Hebei Medical University, Shijiazhuang, Hebei, China; ^4^ Department of Neurosurgery, The Second Hospital of Hebei Medical University, Shijiazhuang, Hebei, China

**Keywords:** melatonin, diabetic cognitive impairment, inflammation, gut microbiome, metabolomics

## Abstract

**Introduction:**

Diabetic cognitive impairment(DCI) presents as a central nervous complication of diabetes especially among aging population. Melatonin (MEL) is known for its antioxidant and anti-inflammation effects in neuroprotective aspects. Recent evidence has demonstrated that the gut microbiome plays a key role in DCI by modulating cognitive function through the gut–brain crosstalk. MEL has been shown to modulate gut microbiota composition in diabetic model. However, the underlying mechanism through which the gut microbiome contributes to DCI remains unclear. This study aims to investigate the effect and mechanism of MEL in attenuating DCI in relation to regulating the gut microbiome and metabolomics.

**Methods:**

Cognitive and memory function were assessed by the Morris water maze test, histopathological assessment of brain tissues, and immunoblotting of neuroinflammation and apoptosis. The levels of serum tumor necrosis factor-α (TNF-α) and Interleukin-18 (IL-18) were measured by enzyme-linked immunoassays to reflect the circulatory inflammation level.16S rRNA microbiome sequencing analysis was performed on control mice(db-m group), diabetic mice(db-db group) and MEL-treated diabetic mice(db-dbMEL group). Gut metabolites changes were characterized using liquid chromatography tandem mass spectrometry (LC-MS/MS).

**Results:**

Our study confirmed that MEL alleviated diabetes-induced cognition and memory dysfunction. MEL protected against neuroinflammation and apoptosis in hippocampus of db-db mice. MEL corrected the increased abundance of *Bacteroides* and *Dorea* and the reduced abundance of *Prevotella* in db-db mice. The vast majority of differential metabolites among the three groups were lipids and lipid-like molecules. MEL significantly restored the reduced levels of pyruvate and lactic acid.

**Discussion:**

Our results supported the use of MEL as a promising therapeutic agent for DCI, in which the underlying mechanism may be associated with gut microbiome and metabolomics regulation.

## 1 Introduction

As diabetes mellitus’s central nervous system complication, cognitive impairment gradually aggravated with the aging of diabetic patients. Dementia is the most severe stage of cognitive impairment (Arvanitakis et al., 2019). The study revealed that among individuals with diabetes, cognitive impairment was observed in 13.1% and 24.2% of those aged 65–74 and over 74 years, respectively (Feil et al., 2011). Based on cross-sectional data, the prevalence of diabetes diagnosed according to ADA criteria among Chinese adults is 12.8% (Li et al., 2020). As China’s population ages, the incidence of diabetic cognitive impairment (DCI) is on the rise. Nevertheless, current strategies for preventing and treating DCI are inadequate.

The intestine is the second brain of the human body, in which the order of magnitude of 10^13^∼10^14^ intestinal microbiota survive, far beyond the total number of somatic cells (Gill et al., 2006). The connection between the gut microbiota and the advancement of DCI through the modulation of cognitive function via the microbiota-gut-brain axis is increasingly supported by research. The CNS influences the balance of the gut microbiota, and conversely, changes in the gut microbiota affect CNS functions through various mechanisms including metabolites, neurotransmitters, immune response, the vagal nerve, and others (Ghaisas et al., 2016; Ansaldo et al., 2019; Bercik et al., 2011). Studies have previously highlighted distinctions in gut microbiota diversity between diabetic patients with and without cognitive impairment (Zhang et al., 2021a).

The hormone Melatonin (MEL, N-acetyl-5-methoxytryptamine) is secreted by the pineal gland and is recognized for its functions in regulating circadian rhythms, as well as its antioxidant and anti-inflammatory effects (Ahmad et al., 2023; Cipolla-Neto et al., 2014; Tu et al., 2021). MEL level *in vivo* is decreased in patients with type 2 diabetes and neurodegenerative diseases such as Alzheimer’s disease (Hardeland et al., 2015). Recent research has illustrated the cognitive enhancement properties of MEL in cases of chronic cerebral hypoperfusion by mitigating oxidative stress, endoplasmic reticulum stress, and apoptosis (Wang et al., 2023; Thangwong et al., 2022). Some studies have suggested a potential link between melatonin (MEL) and the microbiota-gut-brain axis in various animal models (Zhang et al., 2021b; He et al., 2024). However, the precise mechanism through which the gut microbiome contributes to DCI remains unclear. Our previous study demonstrated that MEL mitigates cognitive impairment in diabetic mice through its anti-apoptotic and anti-inflammatory effects on hippocampal neurons (data not shown). The present study aimed to investigate the pharmacological effects of MEL against DCI and elucidate its neuroprotective mechanisms by modulating the gut microbiome.

## 2 Methods

### 2.1 Animals and materials

Twelve-week male specific-pathogen free (SPF) homozygote db-db mice (C57BLKS/J-leprdb/leprdb, n = 20) and age-matched heterozygote male db-m mice with normal blood glucose (C57BLKS/J-leprdb/+, n = 10) were obtained from Changzhou Cavans Laboratory Animal CO.,Ltd (Jiangsu, China). Heterozygous db-m mice were used as controls for homozygous db-db mice. The justification of male rats was based on the relatively longer distance between the urethral orifice and genitals compared with female rats. The animals were housed individually in cages under a 12:12 h light/dark cycle, with temperature maintained at 22°C ± 1°C, and provided *ad libitum* access to food and water. All experimental procedures were carried out in compliance with the Guide for the Care and Use of Laboratory Animals. Approval for animal handling protocols was obtained from the Animal Ethics Committee of Hebei Medical University. A period of 2 weeks was allotted for the animals to acclimate to the laboratory environment before commencing the experiment.

To investigate the impact of MEL, db-db mice were randomly assigned to two groups: db-db (db-db + saline solution, n = 10) and db-dbMEL (db-db + melatonin (20 mg/kg), n = 10). MEL powder (MedChemExpress, USA) was dissolved in anhydrous ethanol and then diluted with physiological saline solution to the required concentration. The db-m mice (n = 10) received saline solution as the db-db group. The drugs were administered intraperitoneally once daily for a duration of 6 weeks. Prior to behavioral assessments, mouse fecal samples were collected in sterile cages, immediately frozen in liquid nitrogen, and stored at −80°C. Behavioral evaluations were conducted following the 6-week drug regimen. Subsequently, the mice were anesthetized using sodium pentobarbital. Hippocampal tissues and serum samples were extracted and preserved in a −80°C refrigerator.

### 2.2 Enzyme-linked immunosorbent assay (ELISA)

Serum samples (n = 5 in each group) were collected for the detection of inflammatory factor concentrations using TNF-α ELISA kit (Proteintech, USA) and IL-18 ELISA kit (Elabscience, China). The test was performed according to the manual instructions.

### 2.3 HE and nissl staining

The brain tissues (n = 5 in each group) were fixed in a 4% paraformaldehyde solution, then embedded in paraffin, and subsequently sliced into 5-μm thick sections of the hippocampus and cerebral cortex. Following this, the sections underwent hematoxylin and eosin staining (HE staining) for 5 min each (Servicebio, China) Nissl staining was carried out with 1% toluidine blue (Servicebio, China), for a duration of 30 min. Post-staining, the sections were washed, dehydrated, and sealed before being examined and imaged using an optical microscope from Zeiss, Germany.

### 2.4 Immunohistochemistry

Brain tissue sections embedded in paraffin were deparaffinized using xylene and then hydrated with descending concentrations of alcohol. Subsequently, the sections underwent antigen retrieval with citric acid buffer and were rinsed with PBS. Following treatment with 3% hydrogen peroxide, the sections were incubated overnight at 4°C with antibodies against caspase-3 and IL-1β. Secondary antibodies were then applied for 30 min at room temperature, and visualization was achieved using DAB. The sections were dehydrated with a series of increasing alcohol concentrations and mounted before being imaged at ×20 magnification. Optical density (OD) values were quantified using ImageJ software.

### 2.5 Morris water maze (MWM) tests and novel object recognition (NOR)

The Morris water maze test is widely recognized as the gold standard for assessing spatial learning and memory in rodents. The experiment was conducted in a circular metal pool measuring 150 cm in diameter and 60 cm in depth, filled with opaque water mixed with skimmed milk powder to obscure a platform placed 1.5 cm below the water surface. The water temperature was maintained at 22°C ± 2°C. The pool was surrounded by distinct visual cues, with the platform positioned at the center of the southwest quadrant, measuring 10 cm in diameter. Mice were released randomly from four starting points (east, west, south, and north) with their heads facing the pool wall. The time taken to find the hidden platform (escape latency) was recorded and analyzed over five consecutive days. Following the removal of the platform on day 6, the time spent in the target quadrant and the number of platform crossings were recorded and analyzed. Behavioral data were captured and analyzed using an automated image acquisition and processing system (SuperMaze software, Shanghai Xinruan Information Technology, Co., Ltd.). Since MWM path varies remarkably, 10 mice in each group were chosen to test avoiding the deviation of results.

The NOR test was performed in a plexiglass white box of dimensions 40 cm × 40 cm × 30 cm. The experiment process was divided into three stages, namely, adaptation, familiarization, and testing. The discrimination index (DI) was used to assess the learning and memory of mice. It was calculated as N/(N + F) × 100%, where N (new) refers to the time to explore the novel object and F (familiar) refers to the time to explore familiar objects.

### 2.6 16S rRNA microbiome sequencing analysis

The fecal bacterial DNA (n = 6 in each group) was extracted for gut 16S rRNA microbiome sequencing and data analysis using the CTAB/SDS method. Invalid data was filtered and excluded by using Flash (Version 1.2.11) and QIIME (Version 1.9.1) software. Then the Clean Tags were obtained for the subsequent analysis. The Clean Tags were clustered as OTU(Operational Taxonomic Units) of a 97% sequence similarity using USEARCH(Version 7.0.1090). The OTUs were classified into various taxonomic levels by comparison with a database. Alpha diversity was computed using Mothur software (Version 1.31.2). Beta diversity was assessed using QIIME (Version 1.80) and visualized through principal coordinate analysis (PCoA). MetaStat and T-tests were employed to detect significantly different gut microbiota species among the three groups at each taxonomic level. The linear discriminant analysis (LDA) effect size, with a LDA score threshold of 2, was utilized to discern significant differences among the groups via the LEfSe software (Version 1.0). According to previous study, the number of samples no less than six is needed to prevent the deviation of standard error.

### 2.7 SCFA extraction and analysis

Fecal samples were mixed with water, centrifuged, derivatized, and analyzed utilizing an ultraperformance liquid chromatography coupled with triplequadrupole tandem mass spectrometry (UPLC-TQ-MS) system (Acquity UPLC-XEVO TQS, Waters Corp., Milford, MA, United States). The results are quantified in micrograms per Gram. One scattered value was removed from the result.

### 2.8 Fecal metabolomics analysis

For the metabolomics analysis, fecal samples were thawed slowly at 4°C in a solution containing methanol, acetonitrile, and water in a 2:2:1 ratio. The samples were then vortexed, mixed for 30 s, and incubated at −20°C for 10 min following sonication in ice water for 30 min. Subsequently, the samples were centrifuged at 4°C for 20 min at 14,000 rpm, and the resulting supernatant was collected for analysis.

Analysis was conducted using a UHPLC system (Agilent Technologies) coupled with a quadrupole time-of-flight (AB Sciex TripleTOF 6600). The mobile phase, comprising phase A (25 mM ammonium acetate and 25 mM ammonium hydroxide in water) and phase B (acetonitrile), was employed in both ESI positive and negative modes. The gradient started at 95% B for 0.5 min, decreased linearly to 65% over 6.5 min, further reduced to 40% in 1 min, maintained for 1 min.

The raw MS data were first converted to MzXML files using ProteoWizard MSConvert and subsequently imported into XCMS software for peak analysis. The processed data underwent analysis through principal component analysis (PCA) and orthogonal partial least-squares discriminant analysis (OPLS-DA) using the R language. The variable importance in the projection (VIP) value of each variable within the OPLS-DA model was computed to assess differential metabolites, with a threshold set at VIP >1 and a *p*-value <0.05 to determine significance. Signal pathway enrichment analysis was performed using the KEGG pathway database.

### 2.9 Statistical analysis

The data were presented as the mean ± standard error and analyzed using GraphPad Prism version 8 (GraphPad Software, La Jolla, CA, USA). Two-way repeated measures analysis of variance (ANOVA) was employed to demonstrate the statistical differences of means within each treatment group at the specific time points for the MWM tests. Group variations were assessed using one-way ANOVA followed by Turkey’s multiple comparison tests. All *p* values <0.05 were considered statistically significant. Spearman’s method was applied to the data for correlation analysis.

## 3 Results

### 3.1 MEL improved spatial learning and memory function in db-db mice

**FIGURE 1:**
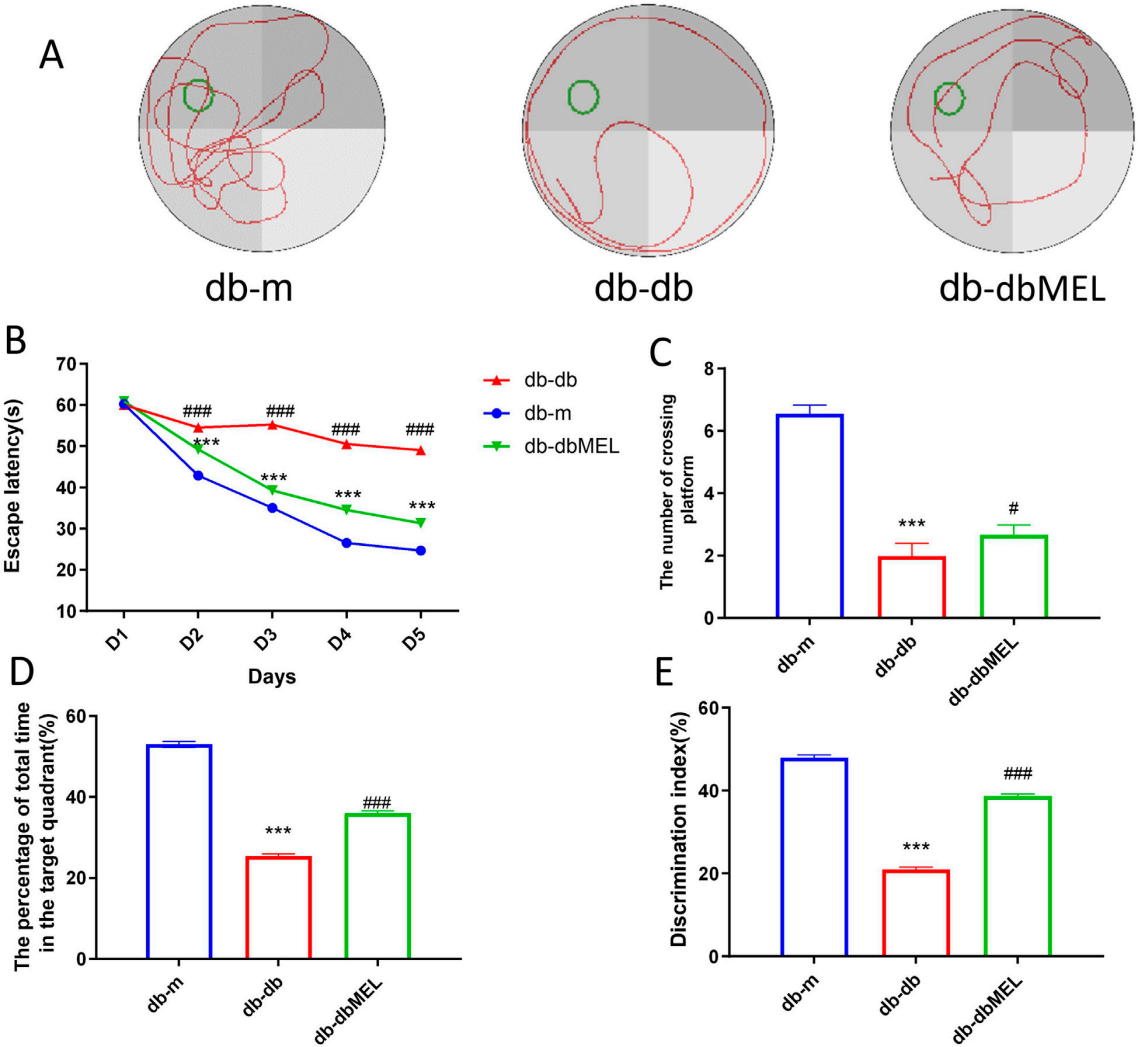
Morris water maze experiment: **(A)** representative swimming trajectories of mice, **(B)** escape latency, **(C)** the number of crossing platform, **(D)** the percentage of total time in the target quadrant, **(E)** discrimination index. All statistical data are presented as mean ± SD, ANOVA analysis. Compared with db-m group: ***p < 0.001, **p < 0.01; compared with db-db group: ^###^ *p* < 0.001, ^#^ *p* < 0.05.

The Morris water maze (MWM) tests were conducted to assess the impact of MEL on cognitive impairment in db-db mice. A notable difference in escape latency time was observed in the db-db group starting from day 2 (54.5 ± 3.0 s) to day 5 (49.0 ± 3.8 s) compared with the db-m group (day 2: 42.9 ± 1.7 s; day 5: 24.7 ± 3.2 s, both *p* < 0.001), which indicated impaired cognitive function in the diabetic mice. Notably, the db-dbMEL group exhibited a significantly decreased escape latency time starting from day 2 (49.2 ± 4.0 s) to day 5 (31.3 ± 1.3 s, *p* < 0.001) compared with the db-db group (Figure 1B). The statistical analysis comparing crossing and swimming times in the platform quadrant on day 5 is illustrated in Figures 1C, D. Mice in the db-db group exhibited significantly shorter platform crossing times (2.0 ± 0.4) compared to the db-m group (6.5 ± 0.3, *p* < 0.001). The db-dbMEL group exhibited a significantly higher number of crossing times compared to the db-db group (2.7 ± 0.3, *p* < 0.05). Likewise, the db-db group demonstrated a significantly lower percentage of time spent swimming in the platform quadrant (53.0% ± 1.0%) in comparison to the db-m group (25.4% ± 0.5%). The db-dbMEL group displayed a significant increase in the percentage of time spent swimming in the platform quadrant (36.0% ± 1.0%) as opposed to the db-db group (*p* < 0.001). Additionally, as illustrated in Figure 1E, a reduced discrimination index was evident in the db-db group (21.0% ± 1.9%) when contrasted with the db-m group (48.0% ± 2.0%, *p* < 0.001). Following MEL treatment, the discrimination index in the db-dbMEL group notably increased (38.7% ± 1.7%, *p* < 0.001). These experimental findings indicate that MEL treatment ameliorated cognitive function decline in db-db mice.

### 3.2 MEL alleviated hippocampal pathological changes in db-db mice

**FIGURE 2:**
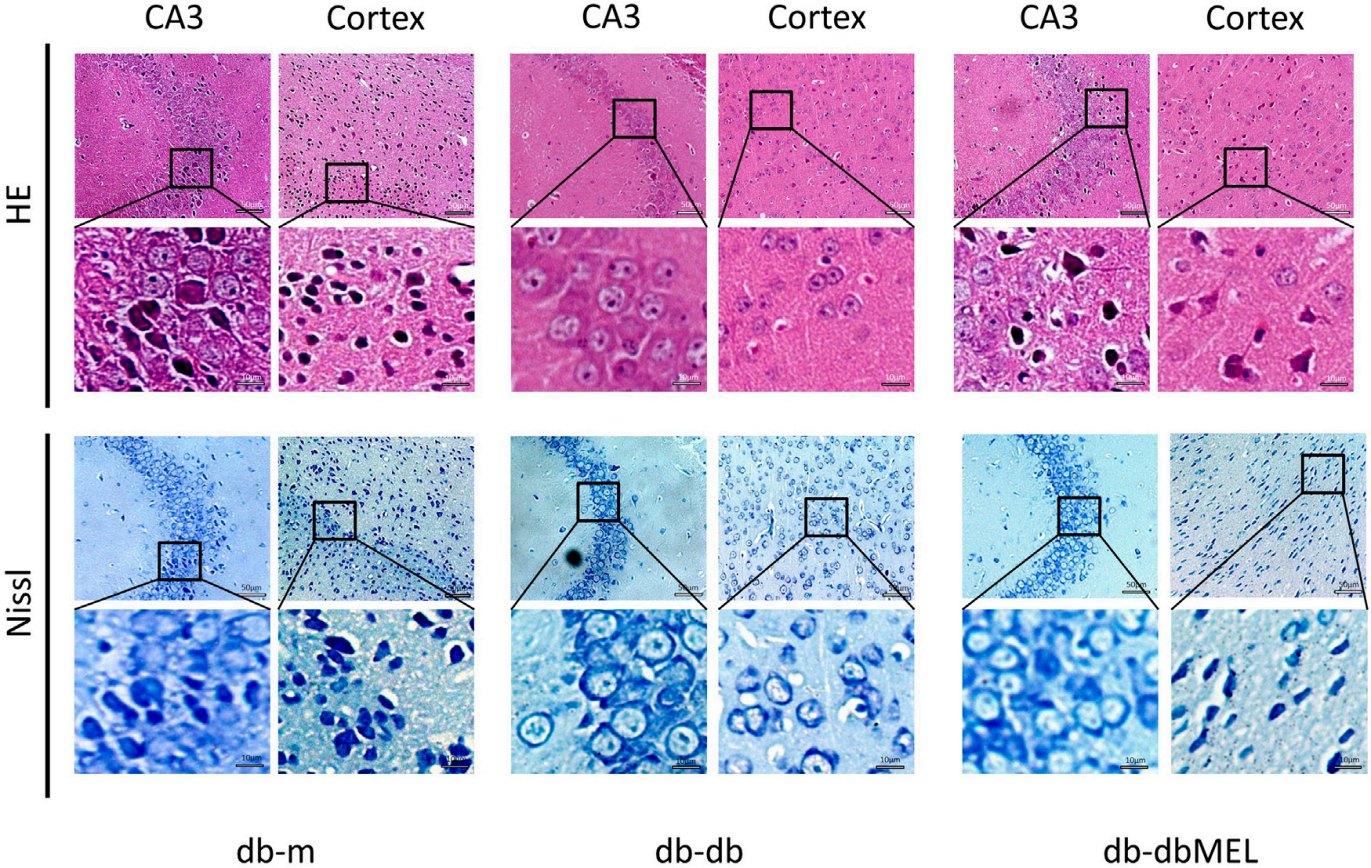
Effect of MEL on histopathologic changes in the mice hippocampal CA3 region and cortex (HE staining and Nissl staining).

Pathological assessment of the CA3 region of hippocampus and the cerebral cortex was performed by hematoxylin and eosin (H&E) and Nissl staining. As shown in Figure 2, the neuronal morphology in the hippocampus and cortex region of db-m mice was clear and evenly distributed, with large and distinct nucleus. In contrast, the db-db group demonstrated pronounced nuclear pyknosis and indistinct nuclei in CA3 and cortex region, which were notably diminished by MEL treatment. Nissl staining indicated a reduction in Nissl bodies, disrupted cellular structure, and neural impairment in the db-db group in contrast to the db-m group. Following MEL intervention, the count of Nissl bodies was restored.

### 3.3 MEL ameliorated inflammation in db-db mice

**FIGURE 3:**
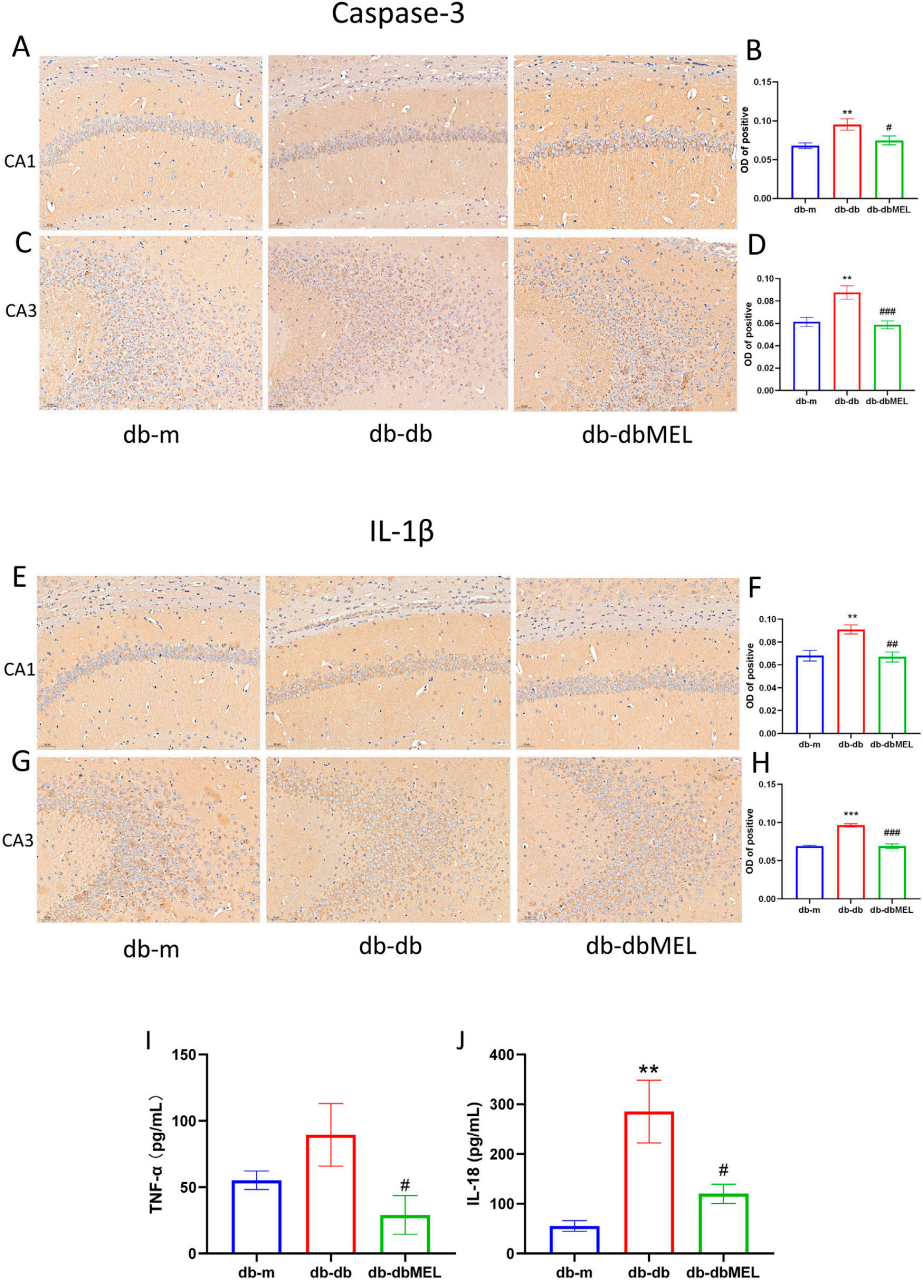
IHC of hippocampus: **(A)** Caspase-3 immunohistochemistry in hippocampus CA1 region of the 3 groups; **(B)** OD analysis of caspase-3 in hippocampus CA1 region; **(C)** caspase-3 immunohistochemistry in hippocampus CA3 region of the 3 groups; **(D)** OD analysis of caspase-3 in hippocampus CA3 region; **(E)** IL-1β immunohistochemistry in hippocampus CA1 region of the 3 groups; **(F)** OD analysis of IL-1β in hippocampus CA1 region; **(G)** IL-1β immunohistochemistry in hippocampus CA3 region of the 3 groups; **(H)** OD analysis of IL-1β in hippocampus CA3 region. **(I)** Serum IL-18 concentration, **(J)** serum TNF-α concentration. All statistical data are presented as mean ± SD, ANOVA analysis. Compared with db-m group: ***p < 0.001, **p < 0.01; compared with db-db group: ^###^ *p* < 0.001, ^##^ *p* < 0.01, #*p* < 0.05.

The study investigated the impact of MEL on inflammatory markers in peripheral blood of db/db mice. Serum IL-18 levels were significantly elevated in the db/db group compared to the db/m group (285.3 ± 62.76 vs. 55.52 ± 10.80 pg/mL, respectively, *p* < 0.001), but MEL intervention notably decreased IL-18 levels in the db-dbMEL group compared with db-db group (120.3 ± 19.31 pg/mL, *p* < 0.01). However, serum TNF-α levels demonstrated no difference between db-db group and db-m group (89.56 ± 23.68 vs. 55.33 ± 6.996 pg/mL, respectively, *p* = 0.0978), MEL significantly reduced TNF-α levels compared with db-db group (29.08 ± 14.57 pg/mL, *p* < 0.05) (Figures 3I, J).

Immunohistochemical analysis (Figures 3A–H) revealed that the optical density (OD) of caspase-3 protein-positive cells in the hippocampus was lower in db/m mice than db-m mice. In contrast, the db/db group exhibited significantly higher OD of caspase-3-positive cells compared to the db-m group (*p* < 0.05). Following MEL treatment, the OD of caspase-3-positive cells in the db-dbMEL group decreased. Similarly, the OD of IL-1β positive cells was notably higher in db-db mice than in the db-m group (*p* < 0.05), with a reduction observed in the db-dbMEL group.

### 3.4 MEL regulated the taxonomy of the gut 16S rRNA microbiome in db-db mice

#### 3.4.1 Diversity

**FIGURE 4:**
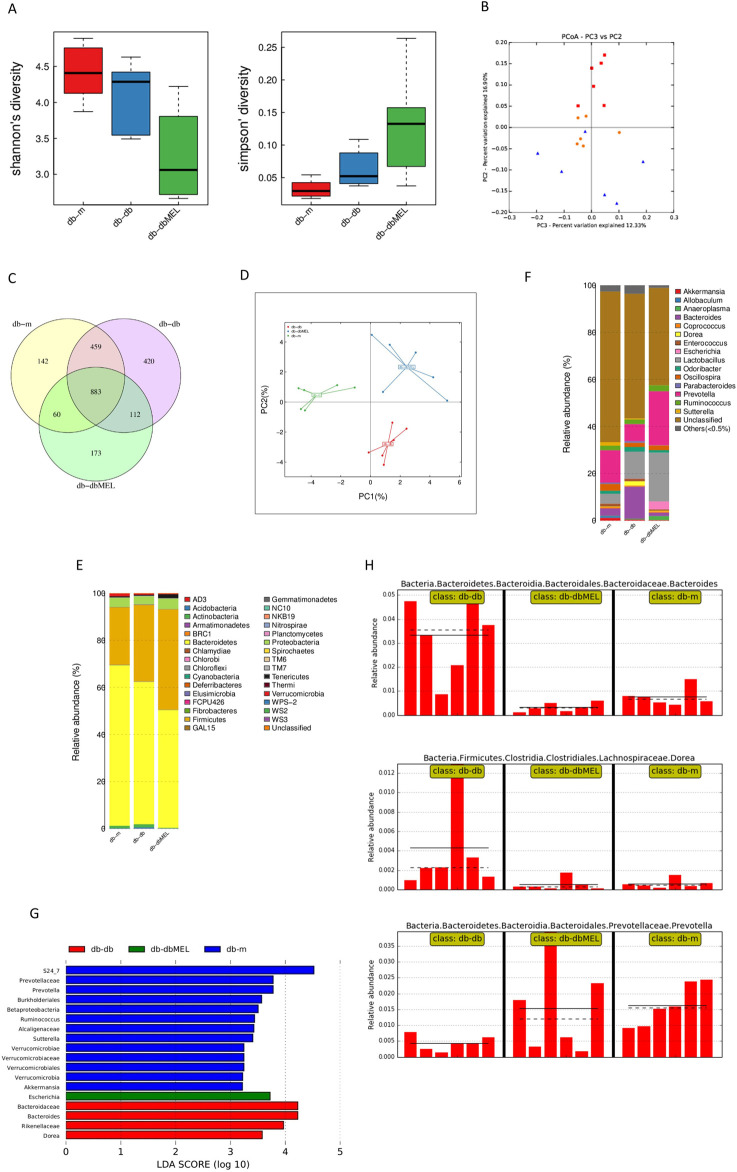
MEL altered gut microbiota composition in db-db mice. **(A)** Alpha diversity index analysis; **(B)** PCA of Beta diversity; **(C)** Venn plots of the OUT abundance of each group; **(D)** principal coordinate analysis (PCoA) of OTU; **(E)** relative abundance of phylum level; **(F)** relative abundance of genus level; **(G)** LEfSe analysis of gut microbiota in each group; **(H)** relative abundance of *Bacteroides*, Dorea, and Prevotella in the gut microbiota based on the LefSe results.

16S rRNA gene sequencing was conducted to assess bacterial composition across three groups. In Figure 4A, microbiome alpha diversity was found to be reduced in db-db mice, indicating a decrease in species variety, but not statistically significant. The Beta diversity analyzed by PCoA, as shown in Figure 4B, suggested that the distribution of the six samples in the db-db group was distinguished from that in the db-m group (principal component 2%: 16.90%, principal component 3%: 12.33%). In contrast, some samples in the db-dbMEL group were distributed similarly to those in the db-m group, indicating that the db-dbMEL group shared similar dominant species with the db-m group, distinct from that of db-db mice.

#### 3.4.2 OTU analysis

The OTU illustrated the gut microbiota community composition among three different groups. The Venn diagram in Figure 4C was performed to utilized to assess the presence of shared and distinct OTUs among these groups.

1342 OTUs were shared between db-db and db-m group, 995 OTUs shared between db-dbMEL and db-db group, and 943 OTUs shared between the db-m and db-dbMEL groups. Additionally, the db-m group exhibited 142 unique OTUs, 420 OTUs in the db-db group, and 173 OTUs in the db-dbMEL group. The PCA analysis at the OTU level showed that gut microbiota structure was changed by MEL treatment in comparisons among the three groups (Figure 4D).

The compositions and taxonomic constitution of the fecal microbiota were assessed at different taxonomic levels, including phylum, class, order, family, genus, and species. At the phylum level, *Bacteroidetes*, *Firmicutes* and *Proteobacteria* were the three most dominant phyla. The ternary plots in Figure 4E showed that the average relative abundance of *Firmicutes* in the db-m group (24.377%) was lower than that in the db-db group (32.465%) and db-dbMEL group (42.652%). The relative abundance of *Bacteroidetes* in the db-m group (68.196%) was higher than that in the other groups (60.494% in db-db and 50.038% in db-dbMEL). The relative abundance of *Proteobacteria* in the db-db group (3.355%) was lower than that in the db-m group (3.953%) and db-dbMEL group (4.442%). At the genus level shown in Figure 4F, *Prevotella*, *Lactobacillus*, and *Bacteroides* were the predominant genera. *Bacteroides* was predominant in the db-db group (13.618%), while *Prevotella* was dominant in the db-m and db-dbMEL group (13.743% and 22.877%, respectively). The relative abundance of *Lactobacillus* in the db-m group (4.232%) was lower than that in the db-db and db-dbMEL groups (11.480% and 20.730%, respectively).

#### 3.4.3 LEfSe analysis

The LDA Effect Size (LEfSe) analysis was applied to compare the differences among the three groups (Figure 4G). At the phylum level, no significant changes were found among the three groups. At the genus level, compared with the db-m group, the relative abundance of *Bacteroides* (Wilcox *p* = 0.004), *Dorea* (Wilcox *p* = 0.009), *Escherichia* (Wilcox *p* = 0.015), were significantly increased, whereas *Prevotella* (Wilcox *p* = 0.002) and *Akkermansia* (Wilcox *p* = 0.041) were decreased in the db-db group. The MEL treatment markedly reduced the levels of *Bacteroides* (Wilcox *p* = 0.002) and *Dorea* (Wilcox *p* = 0.009) in comparison to the db-db group (Figure 4H). The level of *Prevotella* was restored by MEL but not significantly (Wilcox *p* > 0.05).

### 3.5 MEL modulated fecal metabolites profiling in db-db mice

**FIGURE 5:**
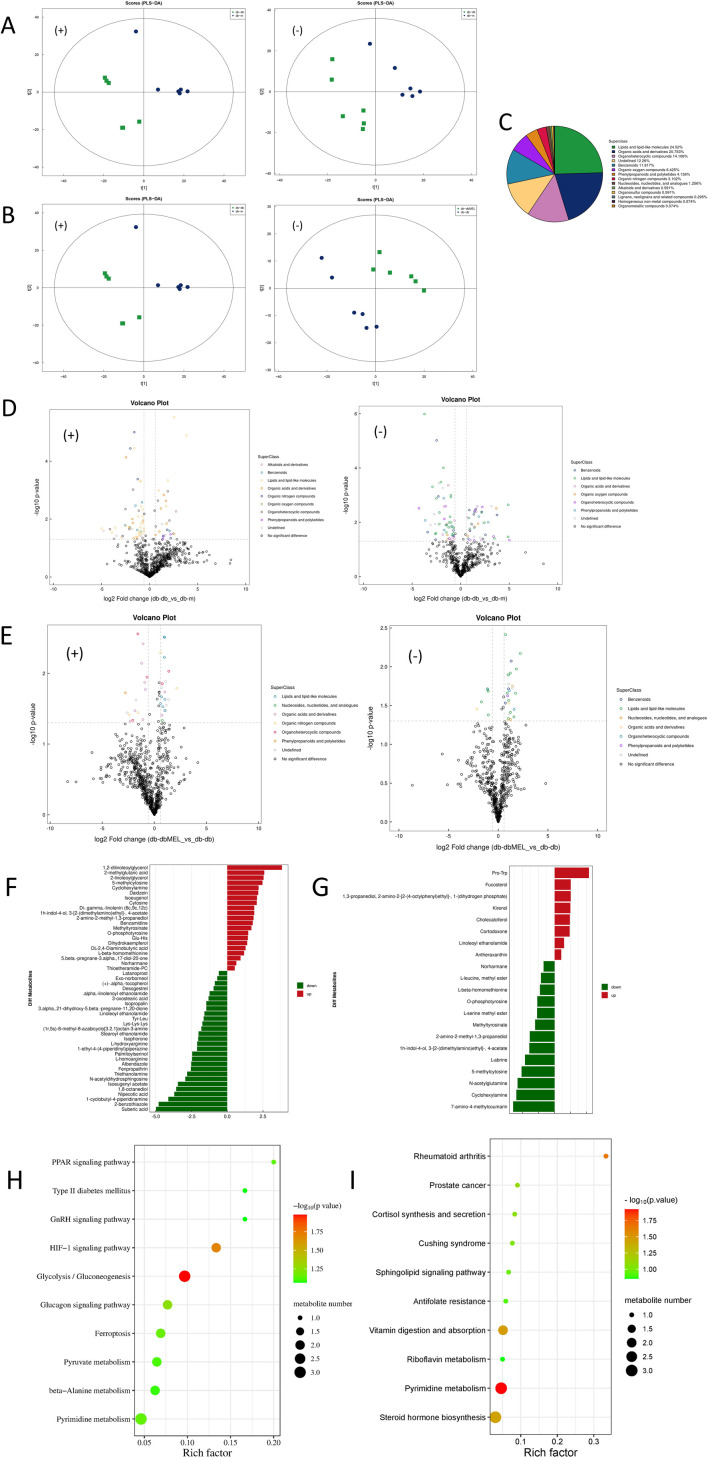
Alterations of fecal metabolites after MEL treatment. **(A)** PLS-DA of fecal metabolites in positive and negative ion modes (compared db-db with db-m group); **(B)** PLS-DA of fecal metabolites in positive and negative ion modes (compared db-dbMEL with db-db group); **(C)** Proportion of the metabolites by chemical taxonomy; **(D)** volcano plots of significant differential metabolites in positive and negative ion modes (compared db-db with db-m group); **(E)** volcano plots of significant differential metabolites in positive and negative ion modes (compared db-dbMEL with db-db group); **(F)** Fold change analysis of significant differential metabolites in positive ion modes (compared db-db with db-m group); **(G)** Fold change analysis of significant differential metabolites in positive ion modes (compared db-dbMEL with db-db group); **(H)** pathway enrichment analysis of differential fecal metabolites (compared db-db with db-m group); **(I)** pathway enrichment analysis of differential fecal metabolites (compared db-dbMEL with db-db group).

PCA, PLS-DA, and OPLS-DA clustering analysis were performed to assess the fecal metabolites Profiling distinction among the three groups. The PCA score plot (Supplementary Material S1) illustrated that the samples of the QC group gathered tightly, which indicated that the results were credible. The PLS-DA scatter plots (Figures 5A, B) showed that both the db-m and db-dbMEL groups were well separated from the db-db group. All the metabolites identified were classified into 14 classes, and the proportion of each class is illustrated in Figure 5C. As shown in the Volcano plots (Figures 5D, E), compared with the db-m group, the db-db group showed 52 differential upregulated and 67 downregulated metabolites. The db-dbMEL group showed 29 differential upregulated and 16 downregulated metabolites compared with the db-db group. The differential metabolites in positive ion modes were shown in Figures 5F, G. The vast majority of these differential metabolites were lipids and lipid-like molecules, such as Suberic acid, Ganoderic acid, 2-cis-4-trans-abscisic acid, Octanoic acid, Pregnenolone sulfate. These differential metabolites showed a lower level in the db-db group compared to the db-m group and exhibited an elevated trend after MEL treatment.

KEGG pathway enrichment analysis revealed that the HIF-1 signaling pathway and Glycolysis/Gluconeogenesis pathway were significantly upregulated in the db-db group compared with the db-m group (Figure 5H). Four metabolic pathways were identified as significantly different between db-dbMEL and db-db groups, including Pyrimidine metabolism, Rheumatoid arthritis, Vitamin digestion and absorption, and Steroid hormone biosynthesis (Figure 5I).

### 3.6 MEl modulated fecal short-chain fatty acid profiling in db-db mice

**FIGURE 6:**
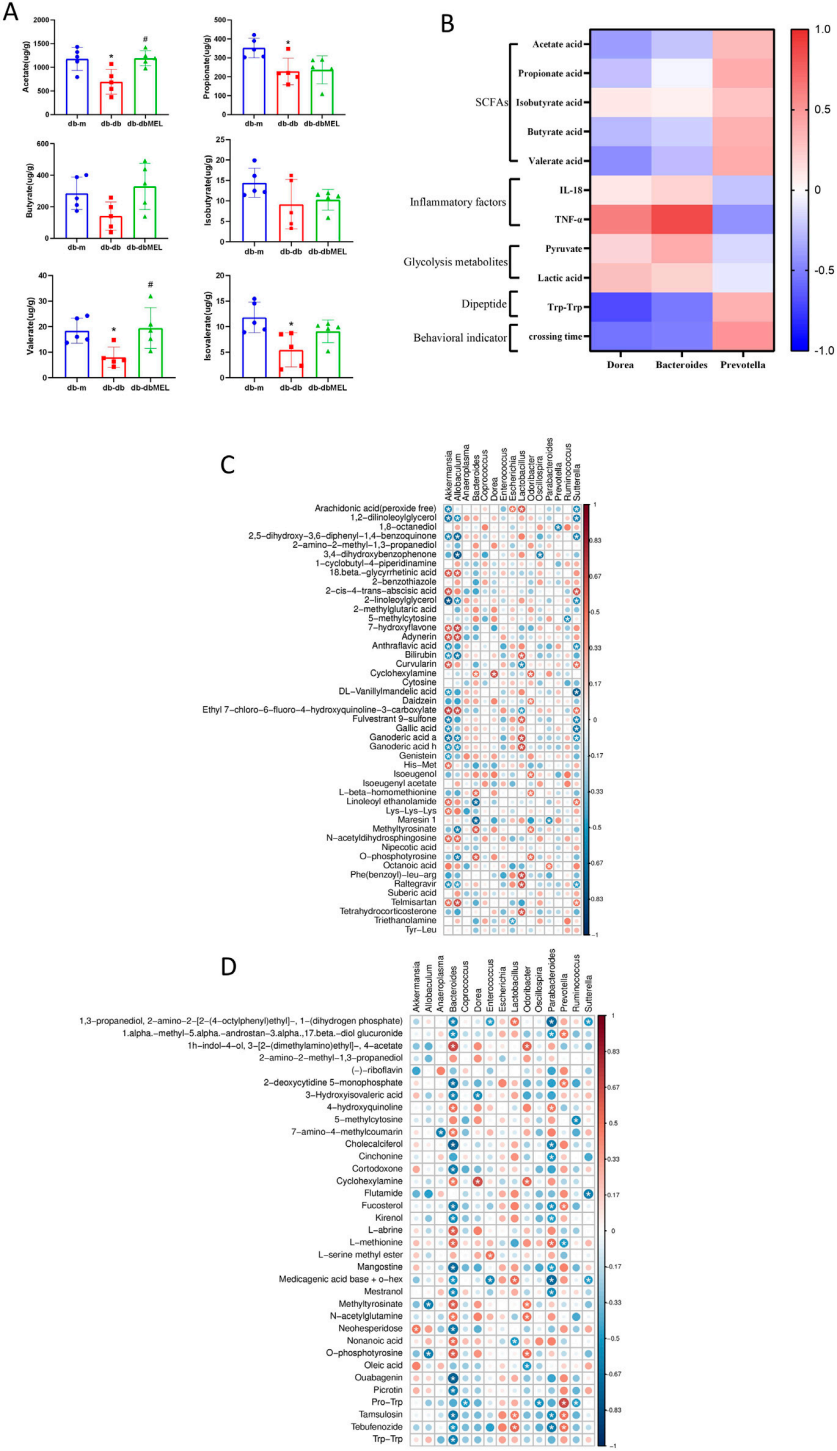
**(A)** Comparison of identified SCFAs in the three groups analyzed by one-way ANOVA test. Compared with db-m group: *p < 0.05; compared with db-db group: ^#^ *p* < 0.05. **(B)** Correlation heat map of changed genera responding to MEL and the DCI-related index, including SCFAs, IL-18, TNF-α, glycolysis metabolites, Trp-Trp, and platform crossing time. **(C)** Spearman’s correlation analysis between the genus level microbiota and differential intestinal metabolites between db-db and db-m group. **(D)** Spearman’s correlation analysis between the genus level microbiota and differential intestinal metabolites between db-dbMEL and db-db group. The red color represents positive correlation, and the blue color represents negative correlation.

Since lipids represented the majority of the differential metabolites, examination of the SCFAs levels in the fecal samples among the three groups was carried out (Figure 6A). Acetate, propionate and butyrate are the three dominant SCFAs in the samples. The level of these three SCFAs was reduced in the db-db group, and the MEL treatment reversed the reduction.

### 3.7 Correlation analysis

The correlations between the key gut microbiota and the biomarkers of DCI including SCFAs, inflammatory factors, glycolysis metabolites, dipeptide, and behavioral indicator were investigated in Figure 6B. Both *Dorea* and *Bacteroides* were significantly downregulated while *Prevotella* significantly upregulated after MEL treatment. *Dorea* and *Bacteroides* were negatively correlated with SCFAs, dipeptides, and platform crossing times. *Dorea* and *Bacteroides* were positively correlated with inflammatory factors and glycolysis metabolites. In Contrast, *Prevotella* was positively correlated with SCFAs, Trp-Trp, and crossing times. *Prevotella* was negatively correlated with TNF-α and pyruvate.

Spearman’s correlation analysis was performed between the genus level microbiota and differential intestinal metabolites. As shown in Figure 6C, *Akkermansia*, *Lactobacillus*, and *Sutterella* were most strongly associated with the vast majority of differential intestinal metabolites between db-db and db-m group. For the differential intestinal metabolites between db-db and db-dbMEL group, *Bacteroides*, *Parabacteroides*, and *Dorea* had a negative correlation with most differential intestinal metabolites (Figure 6D).

## 4 Discussion

Several studies have indicated the beneficial influence of MEL on neurological disease (Liu et al., 2017). The microbiota-gut-brain axis plays a potential role in DCI (Xu et al., 2017). However, the role of MEL in alleviating DCI through the microbiota-gut-brain axis remains unclear. Therefore, it is necessary to explore the potential mechanism by which MEL benefits DCI through the gut microbiota. Our study showed that MEL can alleviate cognitive impairment, neuronal morphology, and inflammation in the hippocampus and serum. These findings are consistent with the findings of the previous study (Cui et al., 2021; Albazal et al., 2021). Our findings indicated that MEL could modulate the composition of the gut microbiota and microbiota-derived metabolites to exert neuroprotective effects. The abundance of *Bacteroides, Dorea*, and *Prevotella* increased after MEL intervention, which may be the key microbiota involved in the improvement in DCI caused by MEL.

The inflammatory response in the hippocampus plays a crucial role in neurodegenerative disease. MEL can inhibit the NLRP3-caspase-1-IL-1β pathway, thereby reducing the secretion of IL-1β and protecting neurons from postoperative cognitive dysfunction (Zhu et al., 2023). Furthermore, MEL can modulate the Nrf2/Sirt1/HO-1 signaling pathway to enhance antioxidant and anti-inflammatory responses (Ali et al., 2020). In the context of diabetic neuropathy, MEL can suppress oxidative stress mediated by glial cells, thus mitigating neuroinflammation. Pro-inflammatory factors such as IL-18, IL-1β, and TNF-α are widely recognized in neuroinflammation and served as inflammatory markers in our investigation. Following MEL treatment in db-db mice, serum levels of IL-18 and TNF-α were reduced. Additionally, restoration of IL-1β and caspase-3 levels was observed in the db-dbMEL group by immunohistochemical method in hippocampus and cortex. The presence of circulating proinflammatory factors can compromise the integrity of the intestinal and blood-brain barriers, potentially facilitating the entry of gut-derived metabolites, toxins, and pathogens into the brain, thereby triggering neuroinflammation (Agirman et al., 2021).

Our study demonstrated that MEL mitigated peripheral inflammation in db-db mice, protecting the gut-brain axis and further exhibiting its anti-inflammation function in the CNS. Previous studies indicate that MEL can alleviate diabetic neuropathy with no effect in lowering blood glucose (Oliveira-Abreu et al., 2021). Although MEL has no significant effect on decreasing blood glucose, the combination of hypoglycemic drugs with MEL may be a new strategy for treating cognitive impairment in patients with diabetes.

Previous studies showed that MEL can promote neurogenesis and reduce neural damage by modulating disturbances in the gut microbiota (He et al., 2024). In our study, the MEL treatment changed the composition of the gut microbiota. The altered microbiota showed associations with behavioral markers and inflammatory parameters. Thus, we speculate that MEL may interact with the gut microbiota to alleviate cognitive impairment through the gut-brain axis. 16S rRNA sequencing revealed an increase in *Bacteroides*, *Dorea*, *Escherichia*, and *Ruminococcus_Lachnospiraceae* abundances, along with a decrease in *Prevotella*, *Ruminococcus_Ruminococcaceae*, and *Akkermansia* abundances in the db-db group compared with those in the db-m group. Moreover, MEL reversed the trend of changes in *Bacteroides*, *Dorea*, and *Prevotella*, indicating that MEL has a neuroprotective effect through modulating the abundance and constitution of the gut microbiota. Several *Bacteroides* species aggravate hippocampal neurogenesis by inducing neuroinflammation (Zafar and Saier, 2021; Wang et al., 2024). Through secreting several neurotoxins, *Bacteroides fragilis* disrupts the intestinal barrier and blood-brain barrier (Lukiw, 2016). Moreover, it leads to microglia activation and cognitive impairment in neurodegenerative diseases (Xia et al., 2023). Previous studies reveal a negative correlation between *Dorea* and cognitive level (Liu et al., 2019; Li et al., 2019). In a rat model of diabetes, an exacerbation of cognitive impairment coincided with an increase in Dorea and a decrease in *Akkermansia* (Zhou et al., 2022). Elevated levels of *Prevotella* are commonly associated with improved cognitive performance (Fongang et al., 2022; Hatayama et al., 2023). A recent investigation illustrated that the transplantation of *Prevotella copri* could alleviate neurological deficits and confer an antioxidant effect in mice afflicted with brain injury (Gu et al., 2024). These studies indicated a positive correlation between cognitive function and *Prevotella,* as well as a negatively correlation with *Bacteroides* and *Dorea.* Furthermore, MEL was found to reverse the abundance of these genera in db-db mice.

Previous studies demonstrated that gut microbial-derived metabolites play a key role in the gut-brain axis. To further assess the metabolic effects of MEL treatment, untargeted metabolomic profiling was conducted in mouse fecal samples. Metabolomics intuitively reflect the metabolic conditions of organisms in different physiological states. Metabolite changes could be a potential mechanism in the progression of disease and a drug target for treating diseases. In our investigation, it was observed that MEL exhibited a specific impact on intestinal metabolites, thereby reinstating the altered metabolites in the db-db group. KEGG enrichment analysis suggested that the main altered signaling pathways in db-db mice were HIF-1 pathway and Glycolysis/Gluconeogenesis pathway, which were closely correlated with energy metabolism. In hypoxic conditions, HIF-1 induces the upregulation of glycolytic pathways to decrease oxygen consumption and sustain essential cellular functions (Kim et al., 2006). In a prior investigation, it was demonstrated that diabetic mice exhibiting cognitive deficits displayed elevated lactate levels alongside reduced choline and energy metabolism (Tiong et al., 2019). Patients suffering from Alzheimer’s Disease exhibited an increased brain lactate concentrations, suggesting the positive correlation between lactic acid level and cognitive decline (Liguori et al., 2015). In our results, the pyruvate and lactic acid produced in anaerobic glycolysis were significantly increased in the db-db group and reversed by MEL treatment. Therefore, we implied that MEL relieves the neural damage under hypoxia condition in diabetes, thereby ameliorating cognitive impairment.

Compared to the db-m group, the levels of short peptides like Trp-Trp, Gly-Tyr, and Tyr-Leu in fecal metabolites were significantly lower in the db-db group. Treatment with MEL reversed these changes, particularly in the case of Trp-Trp. These short peptides, derived from protein proteolysis, are predominantly absorbed directly by the small intestine’s epithelial cells, with the remainder being absorbed and metabolized by gut microbiota for subsequent amino acid metabolism (Daniel et al., 2004). In the colon, proteins are cleaved by various bacterial peptidases, proteases, and endopeptidases to release free amino acids and short peptides, which then subjected to fermentation (Yadav et al., 2018). In recent years, studies show that dipeptides play a biological role in neurological progress in addition to being further metabolized into SCFAs, nitrogenous metabolites, and gases. Pro-Hyp and Tyr-Leu are considered as anti-depressant active peptides that can promote the proliferation of hippocampal neural progenitors (Nogimura et al., 2020; Mizushige et al., 2020). The suppression of microglia by Leu-His demonstrates potential for ameliorating depression-related mood disorders in mice (Ano et al., 2019). Several studies have demonstrated that dipeptides enhance the enrichment of beneficial intestinal bacteria such as *Prevotella* and other SCFA-producing gut microbiota (Sperk et al., 2021; Xu et al., 2021). These coincide with the results of our correlation analysis (Figure 6B). The reciprocal relationship between dipeptides and the intestinal microbiota is evident from these findings. The supplement of dipeptides not only provide nutrients absorbed in gut but also exert its neural activity through the gut-brain axis. Elevated levels of short peptides may represent the underlying mechanism by which MEL regulates the gut microbiota, ultimately alleviating DCI.

SCFAs are small molecules generated by the gut microbiota and are considered pivotal communication mediators in the gut-brain axis due to their ability to cross the blood-brain barrier (Vijay and Morris, 2014; den Besten et al., 2013). SCFAs exert neuroprotective role in CNS diseases by eliminate inflammation, recovering blood-brain barrier permeability, and promoting hippocampal neural plasticity (Qian et al., 2022; Xu et al., 2017). A previous study suggested that acetate metabolism reduces inflammatory signaling in microglia (Soliman et al., 2012). Acetate also regulates hippocampal synaptophysin levels and improves cognitive functions in mice (Zheng et al., 2021). In our study, MEL significantly restored SCFAs, including acetate and valerate acid. In the db-db group, a reduction was observed in the microbiota linked to SCFA production, including *Coprococcus, Oscillospira, and Prevotella*, comparing to the db-m group. However, treatment with MEL reversed this decline. In the correlation analysis, acetate, butyrate, and valerate acid were negatively correlated with *Bacteroides* and *Dorea*, while showing positively correlation with *Prevotella*. *Prevotella* exhibited a positive correlation with platform-crossing times, while showing a negative correlation with serum IL-18 and TNF-α levels. These findings imply a beneficial impact of *Prevotella* and its metabolites SCFAs, on the amelioration of DCI. Conversely, *Bacteroides and Dorea* displayed contrasting correlations with the aforementioned indicators.

In this study, the therapeutic mechanism of MEL was explored only in animal models, indicating the necessity for further clinical trials to ascertain its therapeutic efficacy in humans. While the impact of the gut microbiota and its metabolites on cognitive impairment was elucidated, the precise molecular-level signaling pathway remains unclear. Therefore, clarifying the molecular pathways and metabolite actions within the microbiota-gut-brain axis is essential, which may help identify new therapeutic targets in individuals with DCI.

## 5 Conclusion

MEL treatment ameliorated cognitive impairment in db-db mice by modulating the gut microbiome composition, decreasing the inflammation level, and alleviating diabetic metabolic disturbances. The genera altered by MEL were identified as *Bacteroides*, *Dorea*, and *Prevotella*.

The cognitive function was positively correlated with SCFAs levels, which provides novel therapeutic agents for DCI treatment. In summary, our study elucidated the plausible protective mechanism of MEL against DCI.

## Data Availability

The data presented in the study are deposited in the NCBI repository, accession number PRJNA1178158.

## Supplementary material

SUPPLEMENTARY MATERIAL S1 PCA of fecal metabolites in positive and negative ion modes.
