# Supplementary figures and images for: Effect of melatonin on gut microbiome and metabolomics in diabetic cognitive impairment
Source: Front Pharmacol. 2024 Nov 21;15:1489834. doi: 10.3389/fphar.2024.1489834 (PMC11619431; doi:10.3389/fphar.2024.1489834)

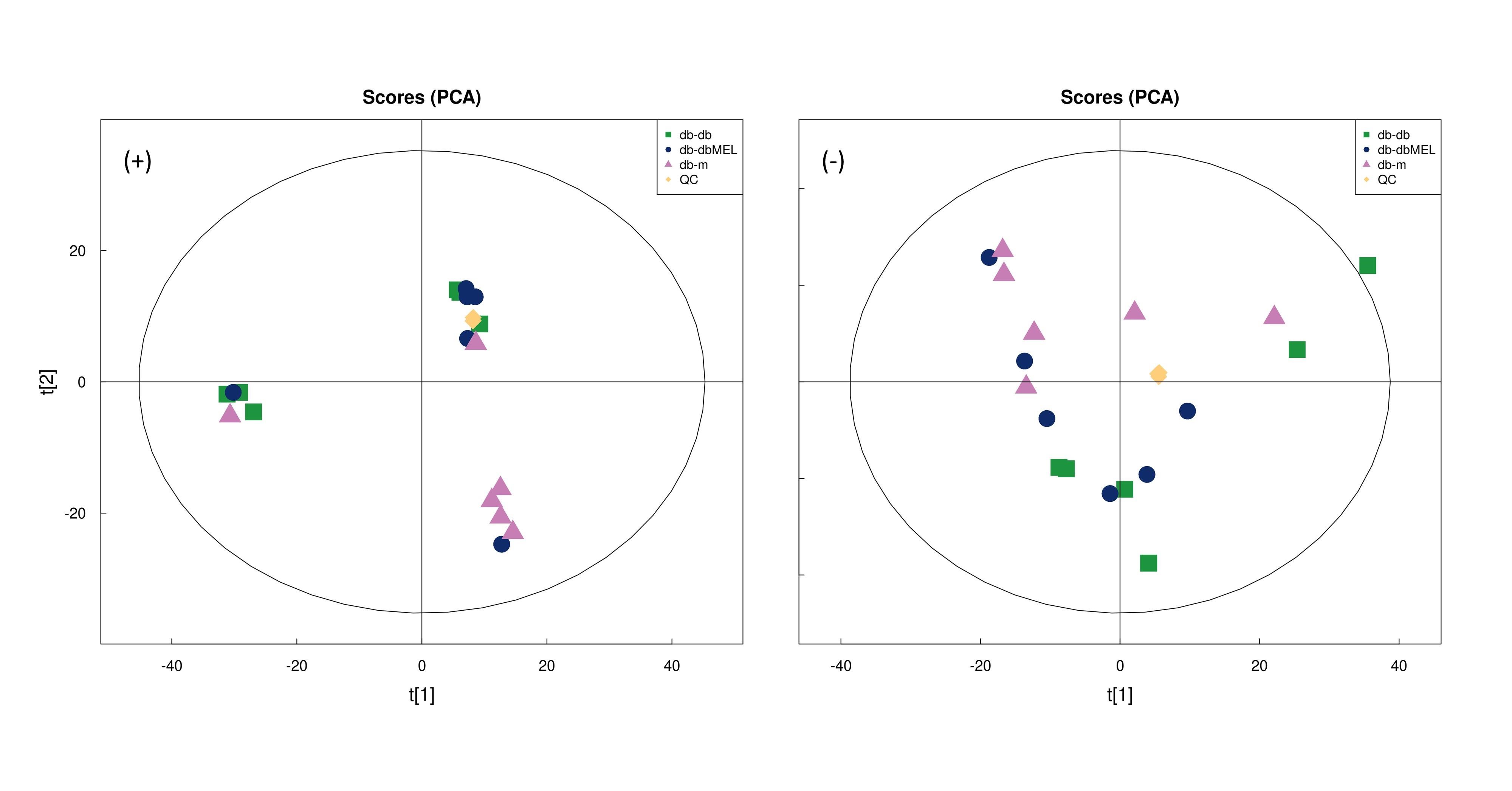

Supplement: Supplementary file 1 [file Image1.JPEG]
